# Novel *RSPH4A* Variants Associated With Primary Ciliary Dyskinesia–Related Infertility in Three Chinese Families

**DOI:** 10.3389/fgene.2022.922287

**Published:** 2022-06-22

**Authors:** Lin Wang, Rongchun Wang, Danhui Yang, Chenyang Lu, Yingjie Xu, Ying Liu, Ting Guo, Cheng Lei, Hong Luo

**Affiliations:** ^1^ Department of Pulmonary and Critical Care Medicine, The Second Xiangya Hospital, Central South University, Changsha, China; ^2^ Research Unit of Respiratory Disease, Central South University, Changsha, China; ^3^ Hunan Diagnosis and Treatment Center of Respiratory Disease, Changsha, China

**Keywords:** primary ciliary dyskinesia, RSPH4A, infertility, asthenoteratozoospermia, MMAF, bronchiectasis

## Abstract

**Background:** The radial spoke head component 4A (*RSPH4A*) is involved in the assembly of radial spokes, which is essential for motile cilia function. Asthenoteratozoospermia in primary ciliary dyskinesia (PCD) related to *RSPH4A* variants has not been reported.

**Materials and Methods:** *RSPH4A* variants were identified and validated using whole-exome and Sanger sequencing in three unrelated Chinese families. High-speed video microscopy analysis (HSVA) was performed to measure the beating frequency and pattern of nasal cilia of the patients and healthy control. Papanicolaou staining and computer-aided sperm analysis were performed to analyze the morphology and motility of the sperm in patient 1. Immunofluorescence was adopted to confirm the structure deficiency of sperm and nasal cilia.

**Results:** Patient 1 from family 1 is a 22-year-old unmarried male presented with bronchiectasis. Semen analysis and sperm Papanicolaou staining confirmed asthenoteratozoospermia. Novel compound heterozygous *RSPH4A* variants c.2T>C, p.(Met1Thr) and c.1774_1775del, p.(Leu592Aspfs*5) were detected in this patient. Patients 2 and 3 are from two unrelated consanguineous families; they are both females and exhibited bronchiectasis and infertility. Two homozygous *RSPH4A* variants c.2T>C, p.(Met1Thr) and c.351dupT, p.(Pro118Serfs*2) were detected, respectively. HSVA showed that most of the cilia in patients 1 and 3 were with abnormal rotational movement. The absence of RSPH4A and RSPH1 in patient 1’s sperm and patient 3’s respiratory cilia was indicated by immunofluorescence. Patient 2 died of pulmonary infection and respiratory failure at the age of 35 during follow-up.

**Conclusion:** Dysfunctional sperm flagellum and motile cilia in the respiratory tract and the fallopian tube were found in patients with *RSPH4A* variants. Our study enriches the genetic spectrum and clinical phenotypes of *RSPH4A* variants in PCD, and c.2T>C, p.(Met1Thr) detected in our patients may be a hotspot *RSPH4A* variant in Chinese.

## Introduction

Primary ciliary dyskinesia (PCD) is an autosomal recessive inherited disease, characterized by motile ciliary dysfunction (Mirra et al., 2017). The clinical manifestations of primary ciliary dyskinesia are varied including chronic upper and lower respiratory tract disease, left-right laterality defects, and infertility (Goutaki et al., 2016). Until now, more than 50 PCD-related pathogenic genes have been identified (Wallmeier et al., 2020).

Each radial spoke dimer contains at least 23 proteins and plays roles in controlling the activity of the dynein arm (Yang et al., 2006). As dynein serves as a molecular motor and the radial spoke complex functions as a mechanochemical senor underpinning the flagellar motility, the disruption of these axonemal components can therefore compromise the motility-related signal transmission and cause asthenospermia (Zhang et al., 2021). The radial spoke head component 4A (*RSPH4A*) is involved in the assembly of radial spokes, which is essential for motile cilia function. The genes belong to the radial spoke complex which is related to PCD including *DNAJB13*, *RSPH1, RSPH9, RSPH3,* and *RSPH4A* (Castleman et al., 2009; Wallmeier et al., 2020). Variants in *RSPH1, RSPH3,* and *RSPH9* genes have been reported to cause male infertility in humans (Reish et al., 2010; Knowles et al., 2014; Jeanson et al., 2015). However, the phenotype of the spermatozoa in patients with *RSPH4A* variants has not been reported.

In this study, we identified three novel *RSPH4A* variants in patients. We first demonstrated asthenoteratozoospermia was related to *RSPH4A* variants and reported two infertile female patients caused by *RSPH4A* variants.

## Case Presentation

**FIGURE 1:**
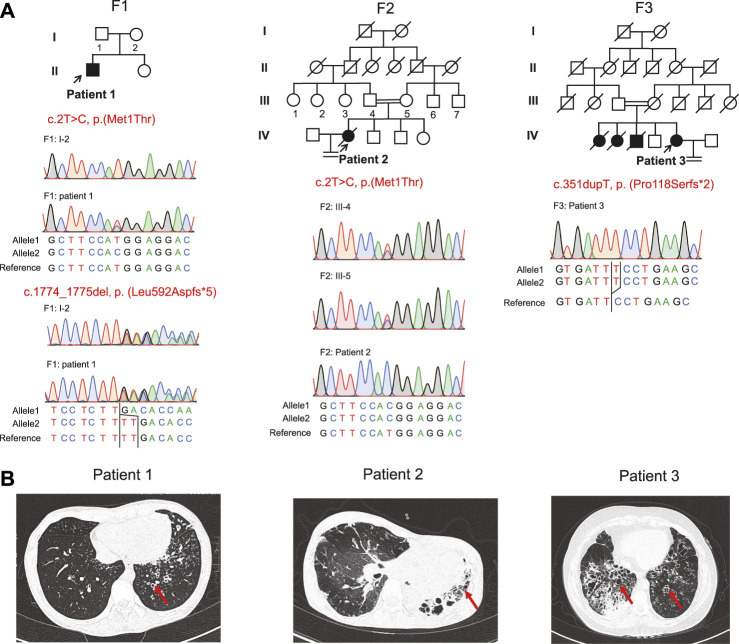
Pedigrees, variants, and clinical features of these patients. **(A)** Pedigrees of three families (F1, F2, and F3) with *RSPH4A* variants and the segregation analysis by Sanger sequencing. The filled symbol represents the affected member, the empty symbol represents unaffected members, and the slashed symbols represent deceased members. Squares, males; circles, females. The patients (patient 1, patient 2, and patient 3) included in this study are indicated by a black arrow. **(B)** Chest high-resolution computed tomography showed bronchiectasis (red arrows) in these patients.

Three patients with *RSPH4A* variants were presented in our study. Patient 1 was a 22-year-old male from a non-consanguineous family I (Figure 1A). Patient 2 was a 34-year-old female from a consanguineous family II. Patient 3 was a 45-year-old female from a consanguineous family III. They all presented over 10 years of history of chronic cough, daily nasal congestion, and yellowish purulent (Table 1). High-resolution computed tomography (HRCT) showed bronchiectasis in all patients (Figure 1B). The nasal nitric oxide level was markedly reduced both in patient 1 (24.6 nl/min) and patient 2 (6.6 nl/min). Patient 1 was unmarried, but the semen analysis showed asthenoteratozoospermia. Both patients 2 and 3 exhibited infertility. Because of the failure to achieve pregnancy, their husbands had completed the fertility examination, and the semen test results were both reported to be normal. Patient 2 died of pulmonary infection and respiratory failure at 35 years of age during follow-up. Two sisters of patient 3 died when they were 1 year old due to repeated pulmonary infection and respiratory failure. One of the older brothers of patient 3 also presented over 10 years of history of chronic cough, daily nasal congestion, and yellowish purulent and died at 37 years of age for the same reason. All family members had no situs inversus reported.

**TABLE 1:** Summary of the clinical features of three patients with PCD.

Characteristic	Patient 1	Patient 2	Patient 3
Sex	Male	Female	Female
Age at diagnosis (years)	22	34	45
Onset	Childhood	Childhood	Childhood
Consanguinity	No	Yes	Yes
Inbreeding coefficient	NA	1/16	1/16
Lung high-resolution CT	Bronchiectasis	Bronchiectasis	Bronchiectasis
Fertility problems	Asthenoteratozoospermia	Yes	Yes
Sinuses CT	Sinusitis	NA	Sinusitis
Smell problem	No	No	Yes
Hearing problems	No	No	No
FEV1% prediction (%)	74.0	NA	45.0
FEV1/FVC (%)	75.0	NA	60.6
Nasal nitric oxide (nl/min)^a^	24.6	6.6	NA
Ciliary beat frequency (Hz)^b^	7.22 ± 1.93	NA	11.44 ± 1.21

NA, not available.

a The nasal nitric oxide of the normal control is greater than 77 nl/min (Collins et al., 2014).

b The ciliary beat frequency of the normal control is 10.08 ± 1.60 Hz.

## Materials and Methods

### Ethical Compliance

This study was approved by the Review Board of the Second Xiangya Hospital of Central South University in China. Written informed consent was obtained from all participants.

### DNA Extraction, Whole-Exome Sequencing, and Variant Calling

We collected blood samples from the patients and their family members, which were obtained with informed consent. According to the manufacturer’s instructions, we used the QIAamp DNA Blood Mini Kit (250) (Cat #51106; QIAGEN, Valencia, CA, United States) to extract genomic DNA. As previously described, whole-exome capture and high-throughput sequencing were performed (Guo et al., 2017). Briefly, the genomic DNA of the patient was captured with the Agilent SureSelect Human All Exon V6 Kit (Agilent, CA, United States) and sequenced on Illumina Hiseq 4000 (Illumina Inc., San Diego, United States). After quality control, the sequencing reads were aligned to the NCBI human reference genome (GRCh37/hg19) by the bwa v7.1.0-r789 (RRID: SCR_010910) (Li and Durbin, 2010). GATK4.0.3.0 (RRID: SCR_001876) was used to call variants, and the variants were annotated with ANNOVAR (RRID: SCR_012821) (Wang et al., 2010; Van der Auwera et al., 2013).

### Variant Filtering and *In Silico* Analysis

We filtered single-nucleotide variants (SNVs) and short insertions and deletions (InDels) as follows: *1*) we ruled out high-frequency variants (minor allele frequency >0.01) in the 1000 Genomes Project data set (RRID: SCR_008801), NHLBI Exome Sequencing Project Exome Variant Server (RRID: SCR_012761), Genome Aggregation Database (all datasets and East Asian population datasets of gnomAD v2.1.1 RRID: SCR_014964 genome database), and the inhouse database of Novogene. *2*) Noncoding and intronic variants were filtered. *3*) Synonymous missense variants were excluded. *4*) Homozygous or compound heterozygous variants were retained *5*) Disease-related variants were identified by a PCD or PCD-candidate gene list collected from the literature (Lei et al., 2022).

### Sanger Sequencing

We adopted Sanger sequencing to validate the variants in the patients. An online tool (PrimerQuest, IDT, https://sg.idtdna.com/PrimerQuest) was used to design primers. The primer sequences used for Sanger sequencing are listed in Supplementary Table S1. PCR products were sequenced by using the ABI PRISM 3730 DNA Analyzer (RIDD: SCR_021899) using the BigDye Terminator v3.1 Cycle Sequencing Kit (Cat #4337454; Thermo Scientific).

### Semen Analysis and Immunostaining of the Sperm and Cilia

Semen sample was collected *via* masturbation from patient 1 after 5 days of sexual abstinence. To obtain the sperm smears, we used a saline solution to wash, centrifuge, and smear spermatozoa from patient 1 and the fertile control individual and fixed in 4% paraformaldehyde. We performed semen evaluation with semen parameters, according to the World Health Organization guideline (Cooper et al., 2010). On the basis of Papanicolaou staining, morphological abnormalities of the flagella were classified as absent, short, bent, coiled, or irregular width (Ben Khelifa et al., 2014). The percentages of morphologically normal and abnormal spermatozoa were assessed according to the WHO guidelines (Sánchez-Álvarez et al., 2010). Immunostaining of the slides was performed, as described previously (Dai et al., 2019). Sperm smears were incubated with primary antibodies: RSPH4A (Cat# HPA031196, RRID: AB_10601612; 1:25; Sigma-Aldrich), RSPH1 (Cat# HPA017382, RRID: AB_1858392; 1:25; Sigma-Aldrich), and anti-acetylated tubulin monoclonal antibody (Cat# T7451, RRID: AB_609894; 1:500; Sigma-Aldrich) for 2 h at 37°C. The binding of the antibodies was detected by incubating with Alexa Fluor 488 anti-mouse IgG (Cat# A-21121, RRID: AB_2535764; 1:400; Molecular Probes) and Alexa Fluor 555 anti-rabbit IgG (Cat# A-31572, RRID: AB_162543; 1:500; Molecular Probes) for 1 h at 37°C. Subsequently, the sperm smears were stained with 4’,6-diamidino-2-phenylindole (DAPI) for 5 min. Finally, they were viewed using an Olympus BX53 microscope (Olympus, Tokyo, Japan) and a scientific complementary metal-oxide semiconductor (sCMOS) camera (Prime BSI; Teledyne Photometrics Inc., United States). Nasal brushing samples from patient 3 were suspended in Gibco Medium 199 (12350039; Gibco). We used an anti-acetylated tubulin monoclonal antibody (Cat# T7451, RRID: AB_609894; 1:500; Sigma-Aldrich) to mark the ciliary axoneme, RSPH4A (Cat# HPA031196, RRID: AB_10601612; 1:100; Sigma-Aldrich) and RSPH1 (Cat# HPA017382, RRID: AB_1858392; 1:100; Sigma-Aldrich) to label the radial spoke in the ciliary axoneme and DAPI to label the nuclei. Immunofluorescence was employed to profile the ciliary structure of patients with *RSPH4A* variants.

### High-Speed Microscopy Analysis

Nasal brush biopsy samples from patients 1 and 3 were suspended in Gibco Medium 199 (12350039 Gibco). An Upright Olympus BX53 microscope (Olympus, Tokyo, Japan) with a ×40 objective lens was used to image strips of the ciliated epithelium. We recorded videos by using a scientific complementary metal-oxide semiconductor (sCMOS) camera (Prime BSI; Teledyne Photometrics Inc., United States) at a rate of 500 frames per second (fps) at room temperature and reviewed at 50 fps to perform an analysis of the ciliary beat pattern, as described previously (Lei et al., 2022). Six separate ciliated epithelium strips (five sideway edges and one from above) with mucus-free regions from the subject were analyzed. The ciliary beat frequency was calculated using the validated automated open-source software CiliarMove (Sampaio et al., 2021).

## Results

### Identification of the *RSPH4A* Variants

Novel compound heterozygous variants c.2T>C, p.(Met1Thr) and c.1774_1775del, p.(Leu592Aspfs*5), a homozygous stat loss variant c.2T>C, p.(Met1Thr), and a novel frame-shift variant c.351dupT, p.(Pro118Serfs*2) in *RSPH4A* (NM_001010892.3) were identified in Proband 1, 2, and 3 through whole-exome sequencing. We did not identify these variants in the 1000 Genomes Project data set and NHLBI Exome Sequencing Project Exome Variant Server, gnomAD v2.1.1 database, and the Human Gene Mutation Database. Sanger sequencing validated the variants in this patient (Figure 1A). These variants were classified into pathogenic or likely pathogenic (Table 2), according to the ACMG guidelines.

**TABLE 2:** RSPH4A variants identified by exome sequencing for the three families.

Family	Gene	HGVS	Amino acid change	GnomAD frequency	MutationTaster	SIFT	PolyPhen-2	CADD	ACMG criterion	ACMG classification
1	*RSPH4A*	N M_001010892.3:c.1774_1775del	p. (Leu592Aspfs*5)	0	NA	NA	NA	NA	PVS1+PM2+PP5	Pathogenic
1	*RSPH4A*	NM_001010892.3:c.2T>C	p.(Met1Thr)	0	Disease-causing	Damaging	Probably damaging	24.0	PVS1+PM2+PM3	Pathogenic
2	*RSPH4A*	NM_001010892.3:c.2T>C	p.(Met1Thr)	0	Disease-causing	Damaging	Probably damaging	24.0	PVS1+PM2+PM3	Pathogenic
3	*RSPH4A*	NM_001010892.3:c.351dupT	p. (Pro118Serfs*2)	0	NA	NA	NA	NA	PVS1+PM2	Likely pathogenic

The variant c.2T>C was found both in patient 1 and patient 2. NA, not available; HGVS, human genome variation society; SIFT, Sorting Intolerant from Tolerant; CADD, Combined Annotation Dependent Depletion score; ACMG, American College of Medical Genetics.

### Motile Cilia Analysis and Immunofluorescence of Nasal Epithelial Cilia

**FIGURE 2:**
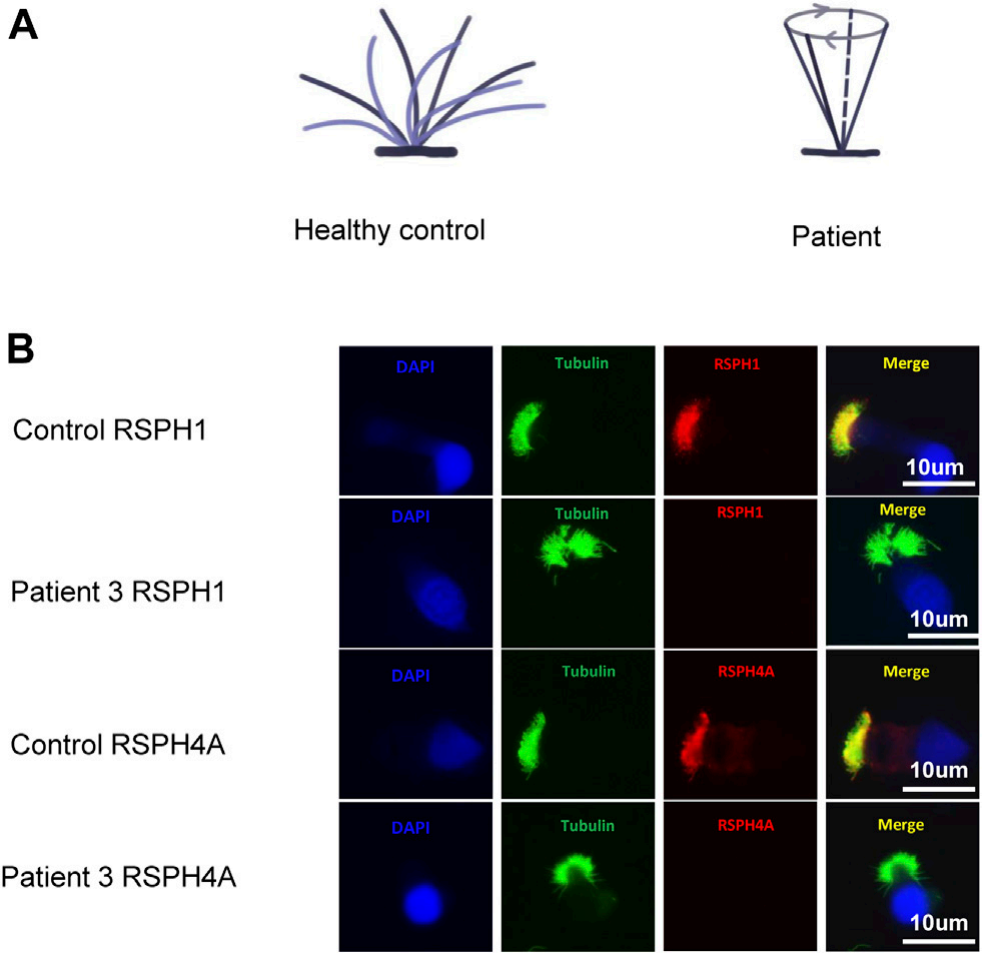
**(A)** Ciliary beating patterns of healthy cilia and our patients with *RSPH4A* variants. **(B)** Immunofluorescence of nasal ciliated cells revealed the absence of RSPH4A and RSPH1 in patient 3 (red). An anti-acetylated tubulin monoclonal antibody was used to mark the ciliary axoneme (green), and DAPI was used to label the nuclei (blue).

Rotational movement of the cilia was observed in patients 1 and 3 (Figure 2A and Supplementary Videos S1 and S2), while planar beating was observed in the healthy control (Supplementary Video S3). High-speed video microscopy analysis of nasal brush biopsy samples indicated that the nasal epithelial ciliary beat frequency of patients 1 and 3 with *RSPH4A* variants was not significantly different from the healthy control (Table1). Immunofluorescence of the nasal epithelial ciliary in patient 3 with *RSPH4A* variants showed both RSPH4A and RSPH1 were absent (Figure 2B).

### Sperm Morphology and Immunofluorescence Analysis

**FIGURE 3:**
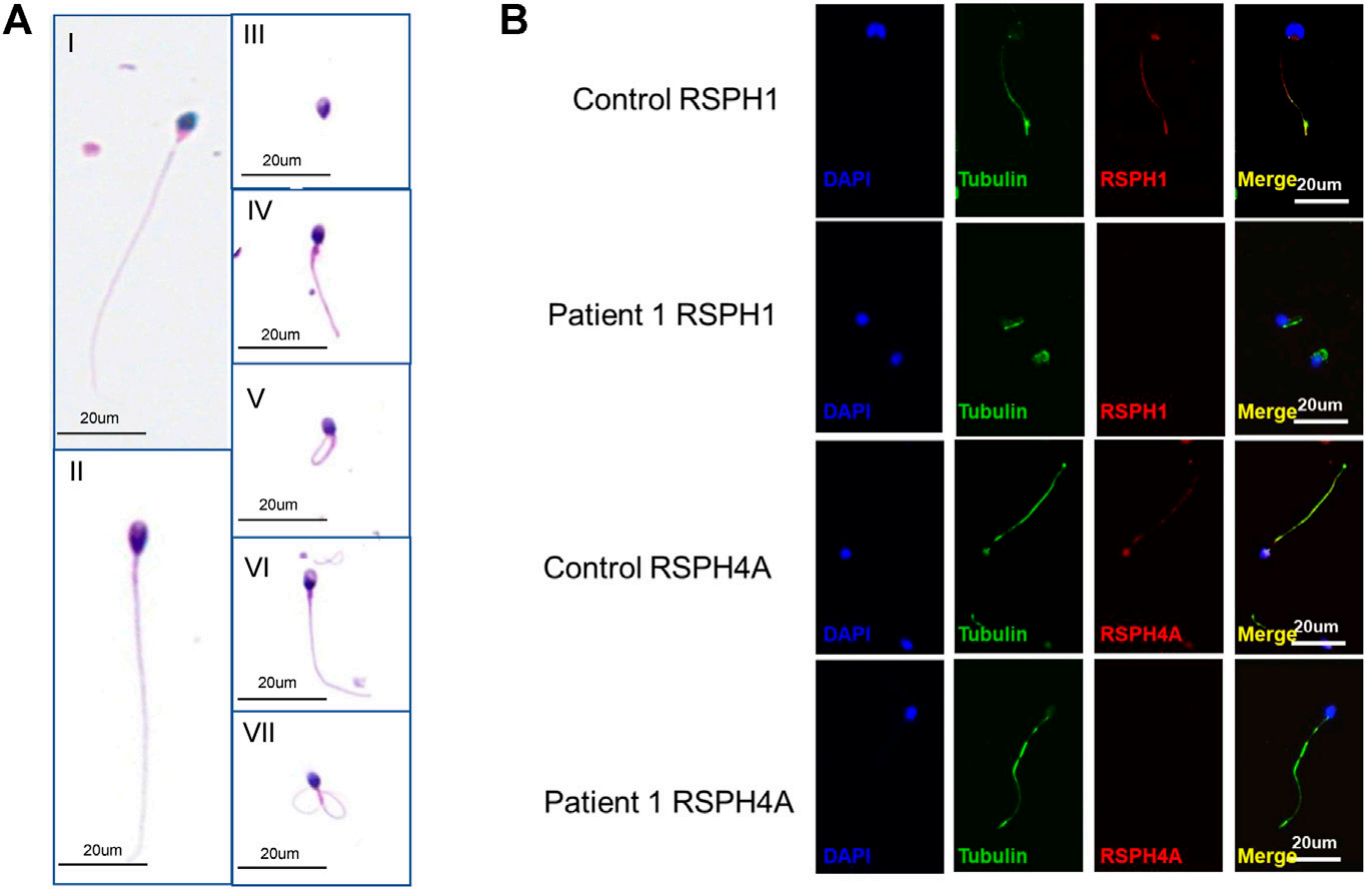
Phenotypic features of the *RSPH4A* mutant sperm and RSPH4A immunostaining in human spermatozoa. **(A)** Papanicolaou staining of spermatozoa from patient 1 and a healthy control. **(A**-I**)** Normal morphology of spermatozoa from a healthy control male. **(A**-II**)** Observed normal length of the sperm from patient 1 with *RSPH4A* variants. In addition, multiple malformations can be observed, including **(A**-III**)** absent, **(A**-IV**)** short, **(A**-V**)** coiled, **(A**-VI**)** bent, and **(A**-VII**)** irregular caliber flagella. **(B)** RSPH4A and RSPH1 are expressed in the whole length of the sperm flagella in the normal control but absent in patient 1.

The abnormalities of sperm morphology were revealed by Papanicolaou staining. Although there were also some spermatozoa with seemingly normal morphology, Papanicolaou staining showed morphological abnormalities in patient 1’s sperm, including absent, bent, coiled, and short flagella, compared with the control individual (Figure 3A). The absent, bent, coiled, and short flagella, respectively, accounted for 11.5, 16.8, 49, and 9.5% in patient 1’s sperm (*n* = 200; Table 3). We conducted semen analysis according to the WHO guidelines (Table 3) and concluded that the patient had asthenoteratospermia given the abnormal morphology of the sperm (Auger et al., 2016). To confirm abnormalities in the axonemal or peri-axonemal structures of the sperm flagella of patient 1, we performed immunostaining. In patient 1’s spermatozoa, the staining signal of RSPH4A was undetectable compared with the normal control (Figure 3B). Moreover, we also could not detect the staining signal of RSPH1 in the sperm of patient 1 (Figure 3B).

**TABLE 3:** Semen parameters and sperm flagella morphology in patient 1 carrying *RSPH4A* variants.

	Patient	Normal control	Reference value
Semen parameters			
Sperm count (10^6/ml)	15.2	54.8	>15.0
Semen volume (ml)	3.0	3.4	>1.5
Motility (%)	49.1	70.1	>40.0
Progressive motility (%)	14.9	40.1	>32.0
Sperm morphology			
Normal flagella (%)	12	50	>23.0
Absent flagella (%)	11.5	7.5	<5.0
Short flagella (%)	9.5	10	<1.0
Coiled flagella (%)	49	15	<17.0
Bent flagella (%)	16.8	7.5	<13.0
Irregular caliber (%)	1.2	10	<2.0

### Summary of Clinical and Functional Phenotypes of Patients With *RSPH4A* Variants

**FIGURE 4:**
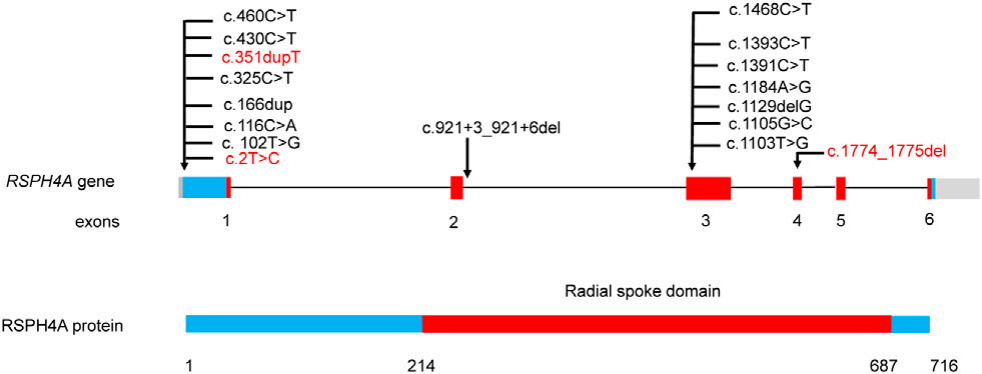
*RSPH4A* gene and protein structure and the reported disease-causing variants of *RSPH4A*. Domains and their relative exons were filled using the same colors. Variants in our study were highlighted using red.

After a comprehensive literature search, 45 patients with *RSPH4A* biallelic pathogenic variants were identified (Ziętkiewicz et al., 2012; Daniels et al., 2013; Casey et al., 2015; Frommer et al., 2015; Emiralioğlu et al., 2020; De Jesús-Rojas et al., 2021) (Figure 4, Supplementary Table S2). About half of the 45 patients with *RSPH4A* variants are Hispanic (21/45), and 15 patients are from consanguineous families. There are 31 females (31/45) and 13 adult patients (≥18 years old, 13/45). Situs inversus is not present in all patients. Only one female patient reported infertility. The nasal nitric oxide production rate was markedly reduced in all 15 patients. A stiff and rotatory beating pattern of HSVA was presented in 11 patients with *RSPH4A* deficiency. Central apparatus abnormalities or normal ciliary ultrastructure were shown in the TEM analysis of respiratory cilia.

## Discussion

This study describes one male and two females with PCD that carried biallelic disease-causing variants in the *RSPH4A* gene. Our cases enrich the variant spectrum and clinical phenotypes of *RSPH4A* variants in PCD.

According to our summary, no situs inversus was reported in patients with *RSPH4A* variants, and the nasal nitric oxide level was low. This is consistent with the clinical manifestations of the three patients in our study. In the *rsph4a* knock-out zebrafish model, there is no situs inversus, which may be related to the “9+0” structure of embryonic nodes without radial spokes, while other motile cilia are of the “9+2” structure (Han et al., 2018). Among the spoke head-related genes, variants of *RSPH4A* lead to the most severe phenotype on the cilia ultrastructure in the PCD patients (Daniels et al., 2013; Knowles et al., 2014). The absence of RSPH4A results in the deficient axonemal assembly of radial spoke head components RSPH1 and RSPH9 (Frommer et al., 2015). Therefore, it is probable that patients with *RSPH4A* variants presented the most serious clinical manifestations among the patients with radial spoke family deficiency. As previously reported, a male patient with the *RSPH4A* variant died at the age of 27 because of respiratory failure caused by pulmonary infection (Bian et al., 2021). In this study, patient 2 also died at the age of 35 because of respiratory failure.

The nasal cilia HSVA of patients with *RSPH4A* variants presented a reduced amplitude and rotatory beating pattern. The HSVA of motile cilia in the airway of the *Rsph4a* knock-out mouse presented rotational movement, which may be caused by the deficiency of radial spokes (Shinohara et al., 2015). The HSVA of our patients 1 and 3 showed rotational movement, and the nasal epithelial ciliary beat frequency was in the normal range. Among previously reported patients, the transmission electron microscope examination of respiratory cilia presented with central apparatus abnormalities and a normal structure. However, because of the COVID-19 epidemic situation, these patients are not able to finish the transmission electron microscope examination. According to European Respiratory Society guidelines (Lucas et al., 2017), we confirmed the diagnosis of PCD by the detection of biallelic variants or a low nasal nitric oxide level combined with the characteristic HSVA presentation of the nasal cilia.

One splicing variant, seven missense variants, two frame-shift variants, and two nonsense variants have been reported (Supplementary Table S2). The most reported variant was c.921+3_921+6del from the Hispanic family, which has been reported in 17 patients. There were two reported *RSPH4A* variants from the Chinese family including c.730+1G>A and c.1454C>G. In our study, three novel variants were identified. Two patients from two unrelated families carried the same variant c.2T>C, which may be the hotspot variant in Chinese patients with *RSPH4A* variants.

Infertility is defined as the failure to achieve pregnancy after 12 months of regular unprotected sexual intercourse (Carson and Kallen, 2021). PCD is a rare inherited disease in which genetic variants impair motile cilia function (Afzelius, 1976). Female and male infertility can be caused by defects in the cilia in the fallopian tubes or in sperm flagella, respectively (Wallmeier et al., 2020). The central apparatus and radial spokes play roles in mechano-chemical sensors to control motility in 9+2 cilia and flagella (Smith and Yang, 2004). The *RPSH4A* gene belongs to the radial spoke family. Therefore, it is persuasive to assume that male patients with *RSPH4A* variants present infertility such as *RSPH1*, *RSPH3*, and *RSPH9* (Castleman et al., 2009; Reish et al., 2010; Knowles et al., 2014).

According to a previous report, three male patients with *RSPH4A* variants were likely to be fertile (Vanaken, 2017). But there was no detailed clinical information, variant descriptions, and phenotype of motile cilia of those patients, and it is still unclear about the fertility status of the male patients with *RSPH4A* variants. Patient 1 in this study presented asthenozoospermia which is one of the main factors contributing to male infertility (Shahrokhi et al., 2020). Male infertility caused by the *RSPH1* or *RSPH9* variant has been clarified (Reish et al., 2010; Knowles et al., 2014). The absence of RSPH4A due to variants in *RSPH4A* results in deficient axonemal assembly of radial spoke head components RSPH1 and RSPH9 (Frommer et al., 2015). Therefore, we have reason to assume that *RSPH4A* variants cause male infertility. Moreover, in a previous report, the sperm of a 21-year-old male with the *RSPH4A* variant was immotile (Bian et al., 2021). According to the semen analysis and sperm morphology, it is probable that the *RSPH4A* variants cause male infertility.

As previously reported, two female patients with *RSPH4A* variants presented infertility, which is consistent with the two female patients in our study. In a previous study, in the *Rsph4a* knock-out mice, the oviduct cilia showed two types of abnormal motion patterns: anti-clockwise rotation and beating with small amplitude, which may cause the failure of oocyte pickup (Yuan et al., 2021) and led to female mouse infertility (Yoke et al., 2020). The reason that female patients with *RSPH4A* variants presented infertility is probably because of the change in the beating pattern of the oviduct cilia. However, because of the difficulty to obtain the clinical sample, we cannot confirm the beating pattern of oviduct cilia in our patients.

In conclusion, our study reported three novel *RSPH4A* variants and first demonstrated asthenoteratozoospermia related to *RSPH4A* variants, which revealed the relationship between *RSPH4A* variants and male infertility. Moreover, we also described the detailed information of two infertile female patients with *RSPH4A* variants. Our study enriches the genetic spectrum and clinical phenotypes of *RSPH4A* variants in PCD and provides more evidence for future genetic counseling and gene-targeted therapy for this disease.

## Data Availability

The original contributions presented in the study are included in the article/Supplementary Materials, further inquiries can be directed to the corresponding authors.
